# Sustaining success: a qualitative study of gay and bisexual men’s experiences and perceptions of HIV self-testing in a randomized controlled trial

**DOI:** 10.1186/s12889-021-12011-0

**Published:** 2021-11-09

**Authors:** Ye Zhang, Rebecca J. Guy, Kirsty S. Smith, Muhammad S. Jamil, Garrett Prestage, Tanya L. Applegate, Damian P. Conway, Martin Holt, Phillip Keen, Benjamin Bavinton, Anna M. McNulty, Colin Batrouney, Darren Russell, Matthew Vaughan, Marcus Chen, Christopher K. Fairley, Andrew E. Grulich, John M. Kaldor, Denton Callander

**Affiliations:** 1 Kirby Institute, UNSW Sydney, Sydney, NSW Australia; 2 Global HIV, Hepatitis and STI Programme, World Health Organization, Geneva, Switzerland; 3 Centre for Social Research in Health, UNSW, Sydney, NSW Australia; 4 Sydney Sexual Health Centre, South Eastern Sydney Local Health District, Sydney, NSW Australia; 5 Thorne Harbour Health, Melbourne, VIC Australia; 6 Cairns Sexual Health Service, Cairns North, QLD Australia; 7 James Cook University, Townsville, QLD Australia; 8 ACON, Sydney, NSW Australia; 9 Central Clinical School, Monash University, Melbourne, VIC Australia; 10 Melbourne Sexual Health Centre, Alfred Health, Melbourne, VIC Australia; 11 Mailman School of Public Health, Columbia University, New York, NY USA

**Keywords:** HIV self-testing, Home HIV testing, Qualitative study, Gay and bisexual men, Sustainability, Australia

## Abstract

**Background:**

HIV self-testing was proved as an effective tool for increasing testing frequency in gay and bisexual men at high risk of infection. Questions remain about understanding *why* HIVST encouraged testing and *how* such success can be translated to programmatic implementation.

**Methods:**

We conducted a qualitative investigation of how FORTH participants experienced and perceived HIVST. Stratified sampling was used to recruit gay and bisexual men participating in the FORTH HIVST intervention to take part in interviews, focusing on infrequent testers and those who had received inaccurate HIVST results.

**Results:**

Our analysis identified several prominent themes organized into two overarching domains from the 15 interviews: (i) aspects of HIVST contributing to HIV testing frequency, and (ii) sustaining HIVST into the future. Participants also believed that their use of HIVST in the future would depend on the test kit’s reliability, particularly when compared with highly reliable clinic-based testing.

**Conclusion:**

HIVST increases the frequency of HIV testing among gay and bisexual men due, in part, to the practical, psychological, and social benefits it offers. To capitalize fully on these benefits, however, strategies to ensure the availability of highly reliable HIVST are required to sustain benefits beyond the confines of a structured research study.

## Background

In 2014, the Joint United Nations Program on HIV/AIDS (UNAIDS) established the ambitious target to diagnose 90% of people living with HIV by 2020 [1], with an estimated one-third of HIV infections globally undiagnosed [2]. Although this global target is not achieved by the end of 2020, UNAIDS and health organizations around the world are gearing up for renewed efforts to achieve an ever more ambitious target of 95% of cases of HIV diagnosed by 2030 [1]. Achieving such high coverage of HIV diagnoses will require considerable investment in HIV testing, including the availability of diverse testing mechanisms to target those most at-risk of infection.

HIV self-testing (HIVST) is a WHO recommended approach to HIV testing that allows individuals to self-collect a specimen (oral-fluid or blood), perform a test, and interpret the results themselves at a time and place of their convenience [3]. Previous reviews have noted that the privacy as well as the convenience afforded by HIVST are salient features that can help reduce structural barriers to traditional, clinic-based HIV testing [4–6]. Various qualitative studies have found high acceptability and willingness to use HIVST among diverse populations at increased risk for HIV, notably gay and bisexual men (GBM) [7–10]. Two clinical trials showed HIVST increased testing among GBM who had never tested before and increased test frequency among those most at risk of HIV, priority populations in Australia [11, 12].

Recognizing the potential of HIVST to extend access to HIV testing as well as increase testing uptake and frequency, in 2019 the World Health Organization (WHO) updated the international guidelines for the use and implementation of HIVST [13]. Since then, about eighty of countries have adopted policies that reference or encourage the use of HIVST to support increased coverage of HIV diagnoses [13]. Even though only a small number of countries currently have approved testing kits available on the market, a great number and diversity of HIVST kits is likely to become commercially available in the coming years [14]. The Australia government approved the first HIVST kit for public sale in late 2018; however, further work is still needed to maximize its uptake among those most at risk of HIV [15, 16].

To support the efforts of Australia and other countries to capitalize on HIVST as a way to improve HIV detection, detailed contextual information is needed on how end-users engage with and view self-testing processes and their potential. Results from randomized trials have shown that HIVST can increase testing frequency among gay and bisexual men [11, 12], it is essential that we understand *why* the kits supported testing and *how* such increases in testing can be sustained beyond a trial context. To that end, we conducted a qualitative investigation of experiences and perceptions of HIVST among GBM in Australia.

## Methods

A qualitative study of gay and bisexual men’s experiences and perceptions of HIVST was conducted, comprising data collected via in-depth, semi-structured interviews conducted in Australia during March and April 2015.

### Parent study

The study presented here was part of a larger study of HIVST, known as Frequency of Rapid Testing at Home (‘FORTH’). FORTH was a non-blinded wait-list randomized control trial of HIVST among gay and bisexual men in Australia conducted during 2013–2017, which evaluated the effectiveness of HIVST to increase HIV testing among GBM, including those with no or an infrequent testing history. Details of this study have been published previously [12]. In total, 362 men participated in FORTH, with eligibility defined as being HIV negative at the time of enrolment, being cisgender men, gay or bisexual, and at ‘high risk’ of HIV infection (defined as reporting any condomless anal intercourse or more than five male sexual partners in the 3 months before enrolment). FORTH participants were provided with four HIVST kits (oral-fluid OraQuick In-Home HIV tests, OraSure Technologies) at enrollment and could order more throughout the study. In addition to the quantitative data collected to evaluate the effects of HIVST, a series of in-depth interviews were held with a sub-sample of study participants.

### Participant recruitment and interviews

At enrollment in FORTH, participants were asked to indicate their willingness to take part in an in-depth interview. From that sub-sample of interested participants and referring to HIV testing data collected in the larger study, stratified sampling was used in order to prioritize interviews with GBM who had different kinds of testing experiences. Specifically, we sought to conduct interviews with two to three participants from each of the following groups, defined as those who: (i) had frequent HIV testing prior to participation-‘frequent tester’ (i.e., those reporting at least one test within the 12 months prior to enrolment), (ii) had infrequent HIV testing prior to participation-‘infrequent tester’ (i.e., those with no test within the 12 months prior to enrolment), (iii) reported using HIVST as their exclusive testing method during the study, and (iv) reported using a combination of HIVST and facility-based testing during the study. Participants from each of these categories were randomly selected to receive an invitation for an interview sent via email; after two attempts of follow-up if contact could not be achieved, further participants were randomly selected and contacted via email. Further, we sought to interview all participants who had: (v) experienced incorrect HIVST results (false reactive, false non-reactive), and/or (vi) all men who underwent HIV seroconversion during the study period.

Interviews followed a topic-based, semi-structured interview guide focusing on: (i) condom use and sexual history prior to using HIVST, (ii) reasons for participating in FORTH, (iii) perceptions and experiences of using HIVST, (iv) changes in testing patterns and sexual behavior as a result of access to HIVST, and (v) the potential of HIVST in the future. The interviews were carried out in person or over the telephone, using a ‘funnel/probe’ technique of in-depth interviewing to move discussions of generalities into specific details and examples relevant to the participant’s experience, including topics not covered in the interview guide but which emerged during the interview [17].

### Data analysis

Interviews were audio recorded, transcribed and verified for accuracy. Any details that could have identified an individual participant were removed from the final transcript and all audio recordings were securely destroyed. Transcripts were imported into NVivo software(version number 12, QSR International, Melbourne, Australia), which facilitates the organization and analysis of qualitative data. Data were analyzed using the techniques of deductive thematic analysis [18], with theme development focused on explanations for why HIVST increased testing frequency and how it could be sustained into the future. The thematic structure was developed iteratively over time through multiple readings of the study data and ongoing collaborative revision between the first and senior authors. In this paper, participant names have been replaced with pseudonyms. Throughout, we have also specified if, at baseline, participants were categorized as an frequent or infrequent tester as per HIV testing frequency prior to participating in FORTH.

## Results

In total, 43 GBM participating in the larger FORTH trial were invited to take part in an interview; two invitation emails were not delivered due to inaccurate contact details with replies received from 25 participants of whom three declined to participate. Of the 20 GBM who expressed willingness to participate in an interview on HIVST, a total of 15 were successfully conducted with the remaining five not completed due to scheduling difficulties. Our final sample of 15 interview participants ranged in age from 32 to 65 years (median age: 44). Participants were identified as frequent or infrequent HIV testers prior to FORTH (7 and 8 men respectively). A total of five participants exclusively used HIVST during the study period, while ten used a combination of facility-based and HIVST. Among those men we interviewed, three had seroconverted to HIV during their participation in FORTH and two received inaccurate HIVST results (false negative). Interviews were conducted after the end of the FORTH study, which means this information was known to both interviewers and participants at the time of their participation in this sub-study.

Broadly, nearly all interview participants described HIVST as an aid in increasing the frequency with which they tested for HIV. As one participant with infrequent HIV testing practices described:

*“Well I test more with the home kits cause before I used to go maybe once a year; now I’m testing every three, four months… it’s better I suppose. The pattern is, is increased, increased frequency.” (Armando, infrequent tester)*

Beyond complimentary results from the quantitative data of FORTH study, themes defined through our analysis were organized into two domains: (i) aspects of HIVST contributing to HIV testing frequency, and (ii) sustaining HIVST into the future.

### Aspects of HIVST contributing to HIV testing frequency

#### Theme 1: practical benefits of HIVST

Every participant highlighted various practical and technical benefits of HIVST, which one described as a “*great intervention product*” *(Jacob, infrequent tester).* In a practical sense, participants commonly highlighted the convenience and privacy afforded by HIVST. Especially among men with infrequent HIV testing practices, convenience was constructed as a primary benefit of HIVST, including flexibility around when and where to test for HIV. As one infrequent tester described*:*

*“Whereas with this home, take-home kit, you know, I can do it any time I want. Any time of the day. You know, wherever it suits me, you know” (Danny, infrequent tester).*

Importantly, the practical features of HIVST were particularly appealing to participants who lived in areas with little or no access to health services, including general practitioners. One participant who lived in a rural area described the challenge of securing an appointment at the closest publicly funded sexual health clinic, which only offered HIV testing once a week. In reflecting on how HIVST could help him and his friends living in the area, he mused:

*“if they could do it (the test) in their own home, then I, definitely, think it would be a lot more safer because then guys would at least be able to know their status whereas a lot of them don’t even know, what to do for testing” (Jarred, frequent tester).*

Similarly, the practicalities of HIVST allowed some participants to overcome some previous barriers to HIV testing, including testing while travelling and during public holidays when traditional services were closed.

Building on the practical benefits of the tests, for most of the men interviewed HIVST seemed to function as a form of ‘*routine enhancement*’, meaning that it provided new opportunities to complement existing testing routines. This capacity did not, for most of the gay and bisexual men we interviewed, replace their existing clinic-based testing, but instead supplemented it, allowing for more frequent testing in most cases. Almost uniformly, participants described the test as easy to use with clear instructions, with the test process itself becoming routine after the first one or two tries (e.g., “*I mean I think, it was pretty straight-forward” (Shawn, infrequent tester*)) . Indeed, some men even drew a direct connection between the ease of HIVST and its practical benefits, describing it as enhancing their HIV testing routine, not replacing it *(*e.g.*,“if I wasn’t able to get into the clinic on, on a day that they’re open, I know that I can still do the home test and then I can still just get into the clinic whenever I next can”(Jarred, frequent tester)).*

#### Theme 2: empowering self-management and fostering responsibility

The second theme relevant to HIV testing frequency among gay and bisexual men was a sense of empowerment provided by HIVST. This significance of HIVST was illustrated by one participant who described it as changing his opinion about HIV testing generally, celebrating the idea that HIVST allowed men to become their *“own doctor” (Jacob, infrequent tester)* and look after their sexual health. As we discuss later, this sense of empowerment was not experienced as positive by all participants but for many it helped create a positive sense of personal responsibility for their HIV status and overall health. Feelings of empowerment and responsibility seemed to also imbue participants with confidence in HIV prevention generally, as one participant explained:

*“I know more about my status and I also know so many ways I … you can protect yourself. You can prevent a lot of things so you’re sort of, “Hang on! I can do this. No worry.” (Jacob, infrequent tester)*

For many participants, there was a clear psychological benefit, like a sense of control over testing, afforded by HIVST that may have also positively affected their approach to HIV testing overall.

As described, men who felt empowered through HIVST also shared feelings of responsibility. This idea of responsibility in the context of HIV was evident in the perceptions and experiences shared by participants who were diagnosed with HIV during the FORTH study period. For two of these three men, their HIV diagnosis came after inaccurate (i.e. false non-reactive results) from an HIVST kit. Specifically, both men used self-testing kits as a precursor for condomless anal sex with new regular partners. They were infected as their partners got the false non-reactive results. Importantly, in both of these cases, the men didn’t *‘blame’* the test for their infection and were explicitly aware of HIVST’s reduced sensitivity during the window period *(George, frequent tester)*. When talking about the feeling of getting a non-reactive HIVST and the resultant infection, the men referred specifically to take responsibility; as one participant plainly said: *“I have to take responsibility for my choices” (George, frequent tester)*. The men did not seize on the opportunity to cast blame for their seroconversion on limitations of HIVST, focusing instead on the role that they played in the outcome and describing the support they received post-diagnosis from their friends and local health facilities.

It should be noted that the responsibility afforded by HIVST was regarded as being double edged, as it could be experienced as both empowering and a burden. While in the minority, some participants described heightened experiences of stress and anxiety when conducting an HIVST for the first time, which did not necessarily diminish with repeated tests. Explanations provided for this stress were not in relation to fears of an HIV diagnosis, but centered instead on concerns about using the HIVST kits correctly. For these men, the responsibility they perceived as conferred by HIVST was, in contrast to most other participants, a new kind of burden. As one man expressed:

*“You could screw up the test and not get the result. I thought it was fine, you know. I mean it’s, it’s, it’s a huge, it’s a huge thing to, for someone to do on their own.” (Greg, frequent tester)*

While the majority of participants situated empowerment and responsibility as positive aspects of HIVST that in some cases seemed to facilitate more frequent testing, it is important to recognize that for others these same psychological effects may have actually undermined their enthusiasm for HIV testing.

#### Theme 3: excitement and altruism: the feelings that motived HIVST

For the majority of participants, HIVST was seen as a part of advancements in HIV prevention and a signal of new opportunities for contributing to the HIV response for the community. Strategies for HIV prevention were characterized by participants as largely unchanged in the period prior to the introduction of HIVST in Australia, so many participants characterized HIVST as a novelty, particularly in a space where novelty was perceived as rare. Indeed, many men attributed the novelty of HIVST as initially motivating their participation in the larger FORTH trial. As one participant put it:

“*I had just genuine interest in how it was, how it all worked and what not. I work in a technical field and this is something that’s close to, to me in that aspect. I wanna see what these things are like.” (Peter, infrequent tester)*

Complementing the perceived excitement about and novelty of HIVST, some participants also described the idea of altruism as another motivation to try HIVST. References to altruism or helping others were occasionally couched in the language of collective responsibility, which included the idea that HIV testing was a way of demonstrating care for community members but also that participation in HIV prevention research (i.e., the FORTH trial) was a way to contribute to the HIV response. Indeed, some participants discussed their motivation for undertaking HIVST as a kind of ‘*giving something back*’ to the community.

*“I was asked if I wanted to participate,”* shared one participant, *“I think it will be good to have some contribution to it. That’s the main thing, yeah. Like they're doing, you're doing, you're doing a study on, on the behavior or, or of the, or if, will it change the behavior of a gay person I suppose.”(Karl, frequent tester)*

### Sustaining HIVST into the future

#### Theme 4: HIVST availability

Beyond the predominantly positive practical, psychological and social benefits associated with HIVST, participants spoke often about the conditions they deemed essential to maintain HIV testing practices into the future. While the majority of participants observed an increase in testing frequency while participating in the HIVST trial there was much less agreement about the degree to which that change would be sustained after the end of the study. The availability of HIVST through the trial was commonly identified as a key factor in driving increases in testing, noting that FORTH participants received test kits delivered directly to their homes. At the time of data collection, there were no HIVST kits available on the Australian market and participants were quick to point out that if such a kit became commercially available, access to it would need to be as easy or easier than access to other modes of HIV testing. Related to this idea, discretion was considered by some men to be as important as access. For example:

*“if you want it to be discreet and things like that, a prescription going to your doctor and things like that it kind of, that’s not a way I’ve seen in my professional practice as a way, or, and I don’t think I would go to my GP to get a script, to get a test from a pharmacy.” (Mark, frequent tester)*

While some participants seemed willing to consider including HIVST in their future testing routines, others reflected on their comfort with traditional clinic-based HIV testing. Many of these men attributed increases to their testing frequency to the ready availability of HIVST kits, and not necessarily to any of the practical, psychological or social benefits described earlier. As one participant explained regarding his anticipated frequency of HIVST in the future:

*“I don’t know whether I would go out and buy one every three to four months (in the future). Maybe if I didn’t have all four given to me and I had to go somewhere to pick them, pick them up, I would only maybe pick up when I’m passing or, or if I wanted to. So I think having the four there does maybe give a bit of bias, bias to me to test because [Yeah] they're sitting there.” (Mark, frequent tester)*

A final point to be made regarding the availability of HIVST relates to the cost of the kits themselves. Participants were asked specifically to speculate about the amount they would be willing to pay for a kit, should they become available for purchase in Australia. Although only a few participants reported that they would be unwilling to pay for a test, the majority cited a price point of $AUD20–40 (~$US15–25) as the most they would be willing to spend per test. Even among men who indicated some willingness to pay, however, it was common for participants to muse about the range of free HIV testing options already available to them, with several stating that the investment in time and money required to purchase an HIVST kit would mean that it was reserved for special or unique circumstances.

#### Theme 5: test accuracy

In imagining a future for HIVST, participants also shared concerns around the test’s reliability, commonly citing the ‘window period’ during which the test’s sensitivity is low. In sharing his plans for the future, one participant described the uncertainty of HIVST in contrast to other modes of testing perceived as more reliable:

*“I kind of felt a little bit like, “Well what's the point? I can get a more accurate response from the clinic that, you know, that's my normal behavior, my normal practice." So that kind of dissuaded me a little bit from, from using it” (Shawn, frequent tester).*

Interestingly, concerns about the reliability of HIVST during window period were offset by some participants who were aware of or involved with trials for HIV pre-exposure prophylaxis (PrEP) that took place in the later stages of FORTH. As one man elaborated:

*“I mean [HIVST] might give you some degree of like what the window period was before but, if you don’t know the sexual history of that person, then it doesn’t really give you that level of confidence. …so, looking at PrEP and PEP programs, and things like that as a, an alternative or something that’s in addition to.” (Phillip, frequent tester)*

## Discussion

Gay and bisexual men in this study shared predominantly positive experiences and perceptions of HIVST, with most believing that their access to the testing kits as part of a research study increased the frequency with which they tested, which is aligned with the finding of several clinical trials in Australia and overseas [11, 12, 19, 20]. Participants commonly described various practical, psychological and social benefits of HIVST as explanations for the increased frequency with which they self-tested. In addition, men also highlighted that for such increases to be sustained in the future, HIVST kits would have to be readily available and highly reliable. As Australia and other countries work towards a target of 95% of HIV cases diagnosed by 2030, strategies that capitalize on these ‘end user’ experiences and perceptions may help drive uptake of HIVST among those who need it the most.

While it is true that men commonly discussed how the perceived benefits of HIVST may have increased their testing frequency, the majority were convinced that the availability of the test kits – delivered directly to their home or collected from a clinic, unbidden and without cost – was a more compelling explanation. This is important because it suggests that the positive effects of HIVST on testing frequency were not all derived from the technical nature of test kits per se but also their ready availability. While the benefits of the tests as supportive of uptake and use are important, the structural components of the clinical trial should not be dismissed (i.e. easily access to free HIVST kits). In reality, despite HIVST kits starting to reach the Australian market in 2018, access to the HIVST in Australia remains rather limited. Currently, Australians have to order the HIVST kits online or through a few specific health facilities, and there are complex regulations before they can order them, ie. watch a short introduction video before the purchase [21]. Indeed, clinics and health agencies seeking to deploy HIVST should consider more advocacy strategies, for example if creating multiple channels for delivery of tests would help more fully realise the potential benefits of self-testing for HIV. A few studies have provided evidence that using mobile technology [22, 23] or peer telephone contact [24] in concert with mailed HIVST kits are feasible and useful strategies for promoting HIVST and linkage to HIV-related care among GBM. Our findings also strengthen the argument that the less expensive access to HIVST is for the end user, the more likely it is to be taken up as an ongoing routine [25].

Despite some participants downplaying the practical, psychological, and social benefits of HIVST, it was clear that for others they were important parts of the test’s overall appeal. Aligning with what has been widely reported in previous research [26–28], participants perceived many benefits of HIVST, which if capitalized upon could address several barriers to HIV testing. Notably, our analysis highlighted the practical appeal of HIVST for people living in regional and remote parts of the country. While many participants were satisfied with their existing clinic-based HIV testing routine, in some parts of the world ready access to clinical services is simply not available. Countries like Australia and the United States grapple with the challenges of HIV infections and diagnoses in regional, remote, and rural areas [29, 30], and migrant populations in countries like China and Nepal face obstacles to accessing clinic-based HIV testing and care [31, 32]; HIVST could play an important role in offsetting issues of access to HIV-related health services [33].

Our finding that HIVST elicited for many gay and bisexual men feelings of empowerment and responsibility suggests one angle for encouraging its future uptake. Similarly, for some men, participating in HIVST research (and HIV testing generally) was motivated by their sense of altruism towards communities of gay and bisexual men. Other research has similarly reported on the importance of empowerment and altruism in the context of HIV testing and HIVST specifically [27]. Marketing agencies and public health officials contemplating campaigns promoting HIVST should consider how to capitalize on these positive features to encourage uptake and use.

Although the findings dominated on the positive aspects of HIVST, it should be acknowledged that its reliability as a diagnostic instrument had both psychological and practical limitations. For many men, the empowerment and responsibility afforded by HIVST proved to be a positive force, but for others it amplified concerns about the test’s accuracy and making a mistake in its administration. The ambivalent effects of intensifying feelings of personal responsibility in this study was consistent with the discussion in previous literatures in relation to HIV testing [34–36]. Regarding successful scale-up of HIVST, the implementors need to consider helping or encouraging men with psychological or emotional burden for testing by themselves to seek support throughout the testing process by a trained provider. In our study, future use of HIVST for some men depends on improvements the accuracy of these tests. We note that the FORTH trial used a test based on oral fluids, whereas the one HIVST currently available in Australia uses blood samples, which are more difficult to use but tend to be more sensitive and specific [37]. There are potential trade-offs between using an easier test versus a more accurate one, particularly when men can otherwise access highly sensitive and specific clinic-based testing. Further, it must be recognized that two participants acquiring HIV in our study as people would use HIVST to assess the risk of condomless sex, which for two men resulted in HIV seroconversion in this study. The oral HIV self-testing kit is still limited by the window period. Any efforts to improve the sensitivity of HIVST or, as suggested by some participants, HIVST programs that are linked with use of HIV PrEP may improve men’s confidence in the self-testing process, which in turn could increase uptake.

Several limitations of this study need to be considered. First, we did not interview individuals who practiced HIVST outside the clinical trial setting in which we provided their free testing kits. Second, all men in our study used an oral fluid HIVST, and the results presented here may not apply when using blood-based fingerstick HIVST, which was subsequently approved for use in Australia. Third, the finding of our study conducted in a setting where has ready access to facility-based HIV testing. The findings may not be directly applicable to other community settings, where access to facility-based HIV testing is poor and HIV self-testing is a matter of necessity rather than choice. Finally, findings are based on a small sample and may not be representative of all participants in FORTH. However, we deliberately selected a sample of interviewees who had diverse patterns of HIVST testing and various prior testing patterns.

## Conclusions

HIVST increases the frequency of HIV testing among gay and bisexual men due, in part, to the practical, psychological, and social benefits it offers. To capitalize fully on these benefits, however, strategies to ensure the availability of highly reliable HIVST are required to sustain benefits beyond the confines of a structured research study. HIVST may be particularly appealing to GBM who do not access traditional, clinic-based options for testing and who are confident embracing a new form of testing. Its utility among these and other populations of MSM will require attention to the ways in which its potential benefits – complemented by practical strategies for delivery and uptake – can be harnessed in Australia and overseas. It should be noted that all men who participated in the present study were ever tested for HIV before. It would be advisable for future studies to examine the views and experiences of HIV self-testing among the first-time testers. In addition, countries seeking to improve HIV diagnosis in affected populations should consider how the potentialities of HIVST can be unlocked with consideration of its practical, psychological, and social dimensions.

## Data Availability

The qualitative interview transcripts are not publicly available due to the data containing information that could compromise research participant privacy.
